# Author Correction: Condensin-mediated restriction of retrotransposable elements facilitates brain development in *Drosophila melanogaster*

**DOI:** 10.1038/s41467-025-56152-x

**Published:** 2025-01-22

**Authors:** Bert I. Crawford, Mary Jo Talley, Joshua Russman, James Riddle, Sabrina Torres, Troy Williams, Michelle S. Longworth

**Affiliations:** 1 Department of Inflammation and Immunity, Lerner Research Institute, Cleveland Clinic, Cleveland, OH 44195 USA; 2 Cleveland Clinic Lerner College of Medicine, Case Western Reserve University School of Medicine, Cleveland, OH 44195 USA

Correction to: *Nature Communications* 10.1038/s41467-024-47042-9, published online 28 March 2024

The original version of this Article contained an error in Fig. 7, in which the legends referring to the data plots in panels a, b, d, and e listed the genotypes as “*eyGAL4,GMRGAL4*>*UAS-gypsyCLEVR, UAS-GFP dsRNA*” and “*eyGAL4,GMRGAL4*>*UAS-gypsyCLEVR, UAS-cap-d3*^*GD913*^ *dsRNA*” rather than “*eyGAL4,GMRGAL4*>*UAS-GFP dsRNA*” and “*eyGAL4,GMRGAL4*>*UAS-cap-d3*^*GD913*^ *dsRNA*”, respectively. This has been corrected in both the PDF and HTML versions of the Article.

