# Supplementary material for: Condensin-mediated restriction of retrotransposable elements facilitates brain development in Drosophila melanogaster
Source: Nat Commun. 2024 Mar 28;15:2716. doi: 10.1038/s41467-024-47042-9 (PMC10978865; doi:10.1038/s41467-024-47042-9)
Supplement: Supplementary file 1 — Supplementary Information [file 41467_2024_47042_MOESM1_ESM.pdf]

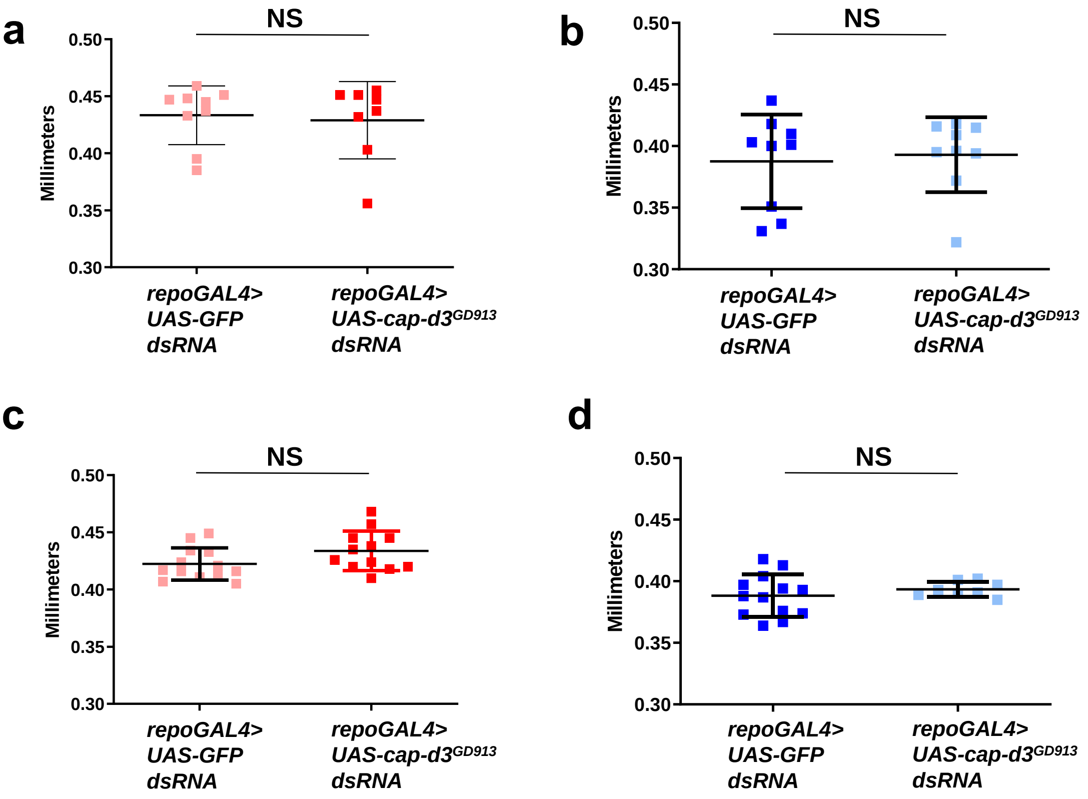
a
NS
Millimeters
0.50
0.45
0.40
0.35
0.30
repoGAL4>
UAS-GFP
dsRNA
repoGAL4>
UAS-cap-d3GD913
dsRNA
b
NS
Millimeters
0.50
0.45
0.40
0.35
0.30
repoGAL4>
UAS-GFP
dsRNA
repoGAL4>
UAS-cap-d3GD913
dsRNA
c
NS
Millimeters
0.50
0.45
0.40
0.35
0.30
repoGAL4>
UAS-GFP
dsRNA
repoGAL4>
UAS-cap-d3GD913
dsRNA
d
NS
Millimeters
0.50
0.45
0.40
0.35
0.30
repoGAL4>
UAS-GFP
dsRNA
repoGAL4>
UAS-cap-d3GD913
dsRNA

Supplementary Figure 1: Cap-d3 knockdown in glial cells and post-mitotic neurons does not affect adult head size. Adult fly head sizes were measured in flies expressing control *UAS-GFP dsRNA* or *UAS-cap-d3* dsRNA, driven by glial cell driver, *repoGAL4* **(a,b)**, or post-mitotic cell driver, *elavGAL4* **(c,d)**. The charts shown are representative of two independent experiments. Measurements from female flies are shown in (a) and (c). Measurements from male flies are shown in (b) and (d). NS= not significant. Error bars indicate standard deviations from the mean**.**

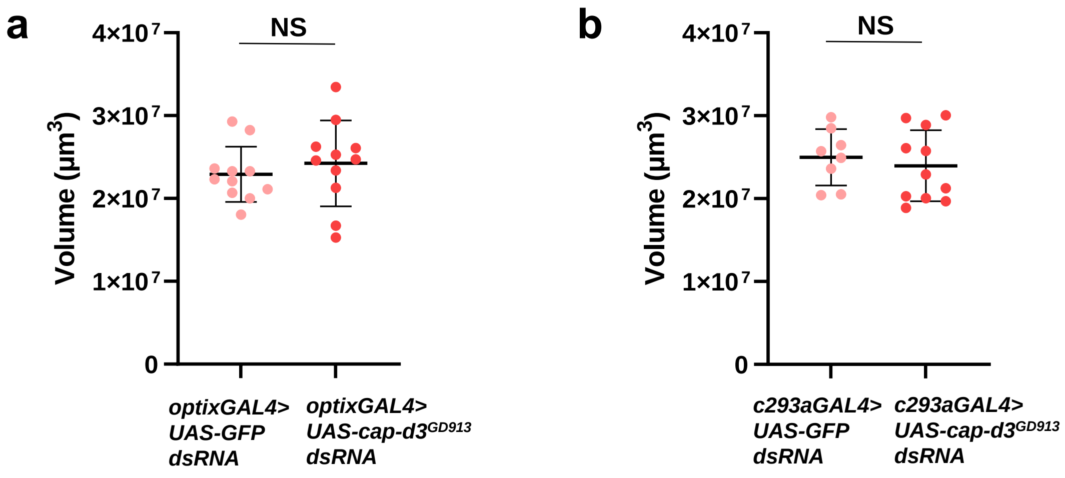
a
b
NS
NS
Volume (µm3)
4×10 7
3×10 7
2×10 7
1×10 7
0
optixGAL4> UAS-GFP dsRNA
optixGAL4> UAS-cap-d3GD913 dsRNA
c293aGAL4> UAS-GFP dsRNA
c293aGAL4> UAS-cap-d3GD913 dsRNA

Supplementary Figure 2: *OptixGAL4* and *c253GAL4* driven expression of *cap-d3 dsRNA* does not significantly affect female adult brain volumes. Adult brain volumes were measured in female flies expressing control *UAS-GFP dsRNA* or *UAS-cap-d3 dsRNA* driven by *optixGAL4* **(a)** or *c293GAL4* **(b)** at 25ºC. The charts shown are representative of two independent experiments. P values were determined by performing Mann Whitney analyses. NS= not significant. Error bars indicate standard deviations from the mean**.**

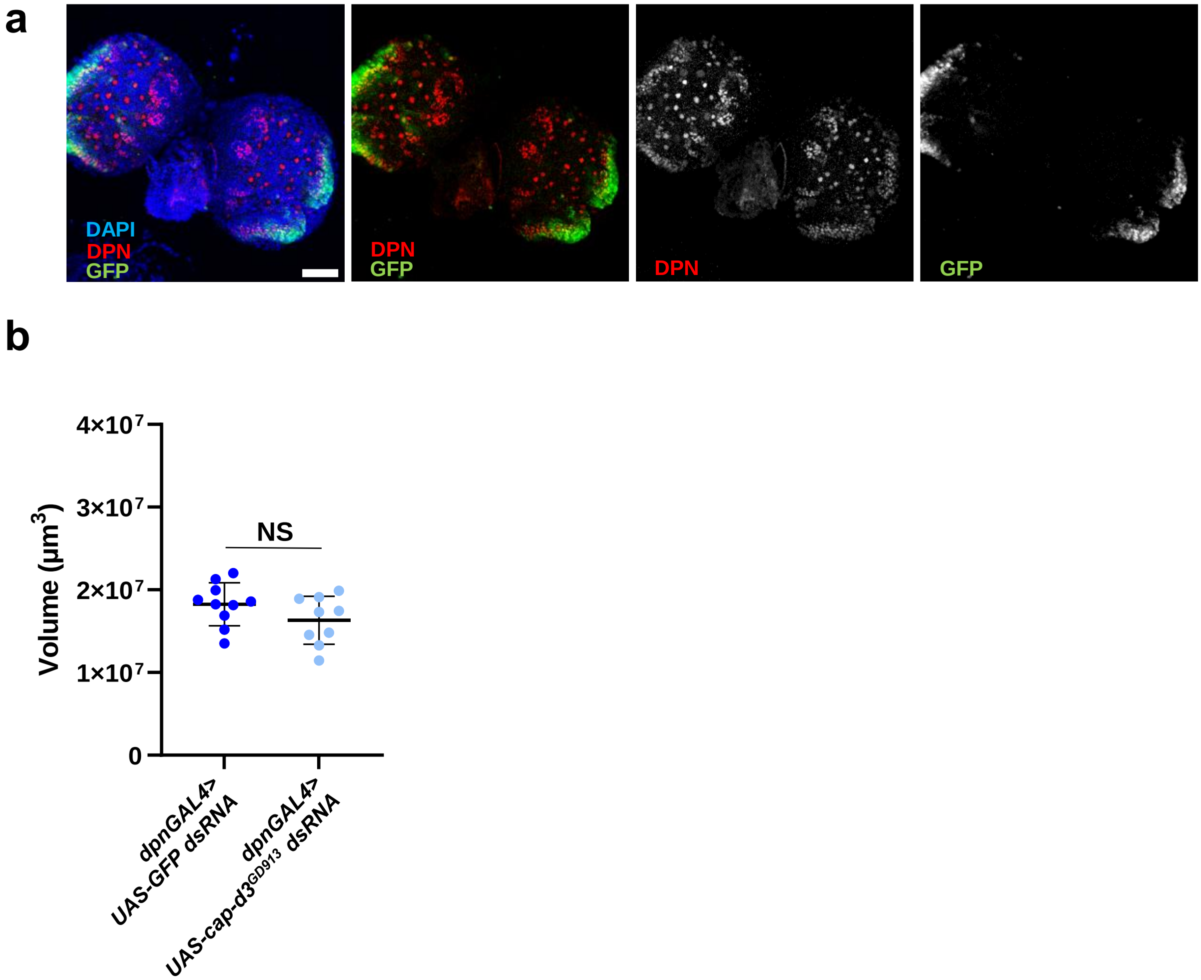
a
DAPI
DPN
GFP
DPN
GFP
DPN
GFP
b
4×10^7
3×10^7
2×10^7
1×10^7
0
Volume (µm^3)
NS
dpnGAL4>
UAS-GFP dsRNA
dpnGAL4>
UAS-cap-d3^GD913 dsRNA

Supplementary Figure 3: Knockdown of Cap-d3 in optic lobe stem cells does not affect adult head size. (a) Third instar larval brains were dissected from male larvae expressing *UAS-GFP* under the control of *dpnGAL4,* immunostained with antibodies to detect GFP (green) and stem cell marker, DPN (red), and imaged using confocal microscopy. Maximum projections of z-stacks are shown. Nuclei are stained with DAPI (blue). All images were taken with 40x magnification. Scale bar= 50µm. Images shown are representative of three biological replicates. **(b)** Adult brain volumes were measured in male flies expressing control *UAS-GFP dsRNA* or *UAS-cap-d3 dsRNA* driven by *dpnGAL4* at 25ºC. P values were determined by performing Mann Whitney analyses. NS= not significant. Error bars indicate standard deviations from the mean**.**

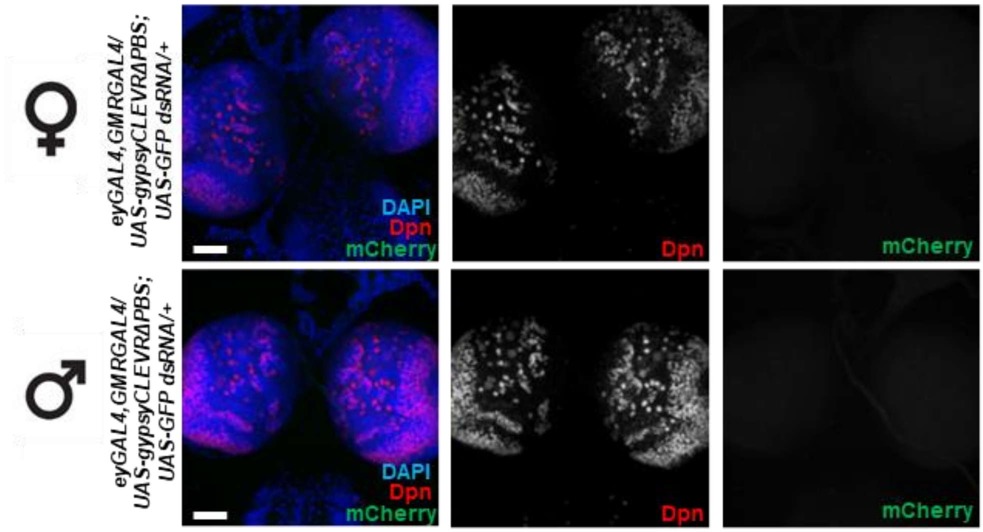
♀
eyGAL4,GMRGAL4/
UAS-gypsyCLEVRΔPBS;
UAS-GFP dsRNA/+
DAPI
Dpn
mCherry
Dpn
mCherry
♂
eyGAL4,GMRGAL4/
UAS-gypsyCLEVRΔPBS;
UAS-GFP dsRNA/+
DAPI
Dpn
mCherry
Dpn
mCherry

Supplementary Figure 4: *GypsyCLEVR* mutants result in the inability to detect retrotransposition in the larval brain. Third instar larval brains were dissected from larvae expressing *UAS-gypsy-CLEVR*$^{\Delta PBS\ 38}$ under the control of *eyGAL4, GMRGAL4,* immunostained with antibodies to mCherry, a reporter of *gypsy* retrotransposition (pseudocolored green), and stem cell marker, Dpn (red), and imaged using confocal microscopy. Maximum projections of z-stacks are shown. Nuclei are stained with DAPI (blue). All images in were taken with 40x magnification. Scale bar= 50µm. Images shown are representative of three biological replicates.

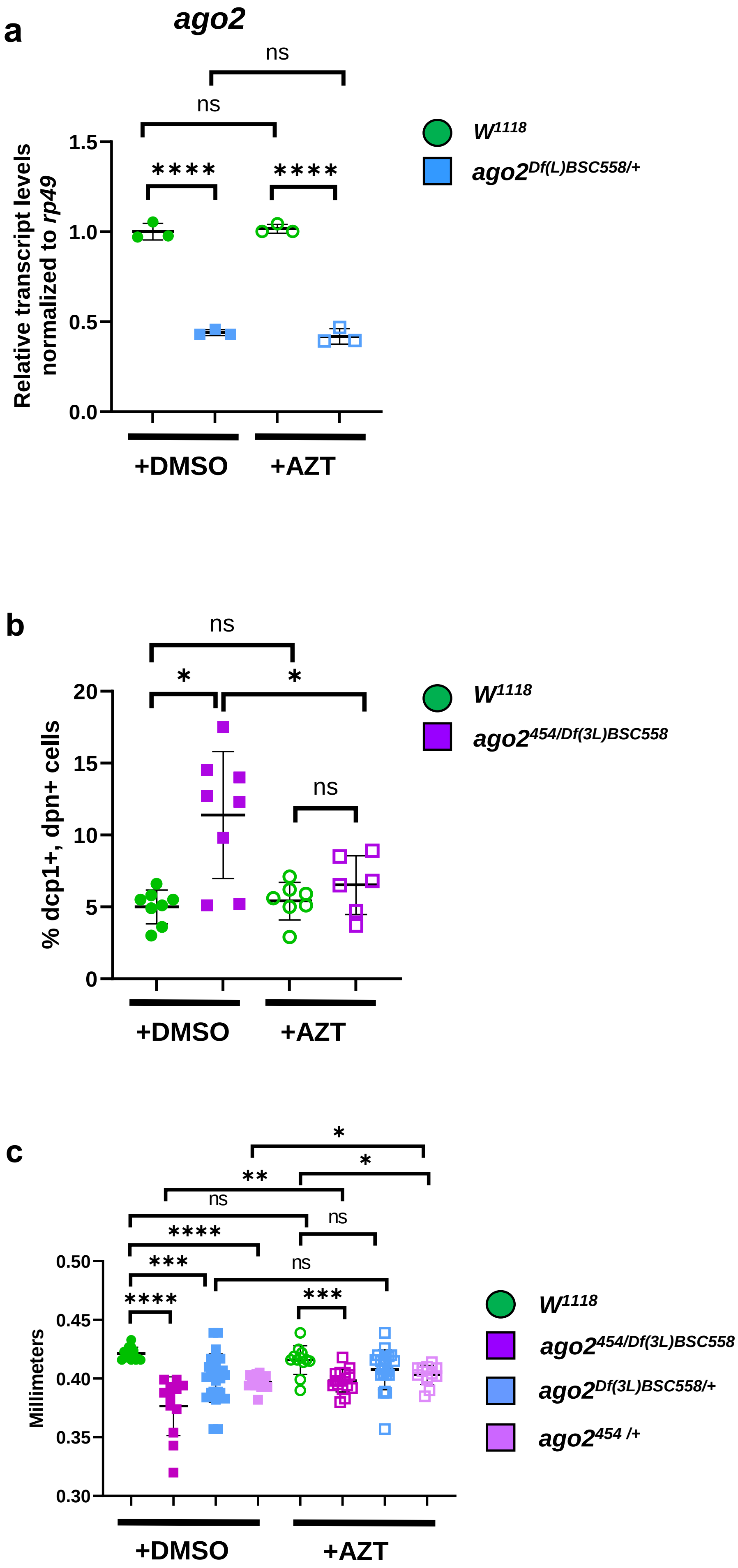
a
ago2
ns
ns
****
****
1.5
1.0
0.5
0.0
Relative transcript levels normalized to rp49
+DMSO
+AZT
W1118
ago2Df(L)BSC558/+
b
ns
*
*
ns
20
15
10
5
0
% dcp1+, dpn+ cells
+DMSO
+AZT
W1118
ago2454/Df(3L)BSC558
c
*
*
**
ns
ns
****
***
ns
****
***
0.50
0.45
0.40
0.35
0.30
Millimeters
+DMSO
+AZT
W1118
ago2454/Df(3L)BSC558
ago2Df(3L)BSC558/+
ago2454 /+

Supplementary Figure 5: NRTIs prevent the increased larval NSPC cell death and microcephaly observed in *ago2* mutants. (a) qRT-PCR analyses of *ago2* transcript levels were performed on cDNA generated from 25 adult female guts harvested from control w^{1118} flies or *ago2* heterozygous mutant flies. Flies were placed on food containing DMSO (black bars) or 5 µM AZT (white bars) for 96 hours, prior to gut dissection. The charts shown include technical replicates. Transcripts were normalized to housekeeping gene *rp49*. P values were determined by performing an unpaired student's T test. **(b)** Third instar larval brains were dissected from wild type, male larvae (green circles) or *ago2* transheterozygous mutant male larvae (magenta squares)**,** immunostained with antibodies to detect stem cell marker, Dpn and cell death marker, Dcp1, and imaged using confocal microscopy. The percentage of cells that stained positive for both Dpn and Dcp1 were quantified. Larvae were developed on food containing DMSO as a control (closed circles and squares), or on food containing 5 µM AZT (open circles and squares). **(c)** Adult male head sizes were measured on wild type flies (green circles), on *ago2* transheterozygotes (magenta squares) or *ago2* heterozygotes (blue and light purple squares). Flies were developed on food containing DMSO as a control (closed circles and squares), or on food containing 5 µM AZT (open circles and squares). For (b, c) P values were determined by performing Mann Whitney analyses. $*p \leq 0.05$, $**p \leq 0.01$, $***p \leq 0.001$, $****p \leq 0.0001$. NS= not significant. Error bars indicate standard deviations from the mean**.**
