# Supplementary material for: Condensin-mediated restriction of retrotransposable elements facilitates brain development in Drosophila melanogaster
Source: Nat Commun. 2024 Mar 28;15:2716. doi: 10.1038/s41467-024-47042-9 (PMC10978865; doi:10.1038/s41467-024-47042-9)
Supplement: Supplementary file 3 — Reporting Summary [file 41467_2024_47042_MOESM3_ESM.pdf]

nature portfolio

Corresponding author(s): Michelle S Longworth

Last updated by author(s): 3/12/2024

Reporting Summary

Nature Portfolio wishes to improve the reproducibility of the work that we publish. This form provides structure for consistency and transparency in reporting. For further information on Nature Portfolio policies, see our Editorial Policies and the Editorial Policy Checklist.

Statistics

For all statistical analyses, confirm that the following items are present in the figure legend, table legend, main text, or Methods section.

n/a	Confirmed	
☐	☒	The exact sample size (*n*) for each experimental group/condition, given as a discrete number and unit of measurement
☐	☒	A statement on whether measurements were taken from distinct samples or whether the same sample was measured repeatedly
☐	☒	The statistical test(s) used AND whether they are one- or two-sided<br>*Only common tests should be described solely by name; describe more complex techniques in the Methods section.*
☐	☒	A description of all covariates tested
☒	☐	A description of any assumptions or corrections, such as tests of normality and adjustment for multiple comparisons
☐	☒	A full description of the statistical parameters including central tendency (e.g. means) or other basic estimates (e.g. regression coefficient) AND variation (e.g. standard deviation) or associated estimates of uncertainty (e.g. confidence intervals)
☐	☒	For null hypothesis testing, the test statistic (e.g. *F*, *t*, *r*) with confidence intervals, effect sizes, degrees of freedom and *P* value noted<br>*Give P values as exact values whenever suitable.*
☒	☐	For Bayesian analysis, information on the choice of priors and Markov chain Monte Carlo settings
☒	☐	For hierarchical and complex designs, identification of the appropriate level for tests and full reporting of outcomes
☒	☐	Estimates of effect sizes (e.g. Cohen's *d*, Pearson's *r*), indicating how they were calculated

Our web collection on statistics for biologists contains articles on many of the points above.

Software and code

Policy information about availability of computer code

Data collection	Volocity imaging software (Quorum Technologies) was used. https://www.volocity4d.com/
Data analysis	Volocity imaging software (Quorum Technologies) was used. https://www.volocity4d.com/. All Data analysis and statistical calculations were performed using Graphpad Prism software, version 10.

For manuscripts utilizing custom algorithms or software that are central to the research but not yet described in published literature, software must be made available to editors and reviewers. We strongly encourage code deposition in a community repository (e.g. GitHub). See the Nature Portfolio guidelines for submitting code & software for further information.

Data

Policy information about availability of data

All manuscripts must include a data availability statement. This statement should provide the following information, where applicable:

- Accession codes, unique identifiers, or web links for publicly available datasets
- A description of any restrictions on data availability
- For clinical datasets or third party data, please ensure that the statement adheres to our policy

Source data are provided with this paper. Further information and requests for resources and reagents should be directed to and will be fulfilled by the corresponding author, Michelle Longworth (longwom@ccf.org).

Research involving human participants, their data, or biological material

Policy information about studies with human participants or human data. See also policy information about sex, gender (identity/presentation), and sexual orientation and race, ethnicity and racism.

Reporting on sex and gender	N/A
Reporting on race, ethnicity, or other socially relevant groupings	N/A
Population characteristics	N/A
Recruitment	N/A
Ethics oversight	N/A

Note that full information on the approval of the study protocol must also be provided in the manuscript.

Field-specific reporting

Please select the one below that is the best fit for your research. If you are not sure, read the appropriate sections before making your selection.

[x] Life sciences [ ] Behavioural & social sciences [ ] Ecological, evolutionary & environmental sciences

For a reference copy of the document with all sections, see nature.com/documents/nr-reporting-summary-flat.pdf

Life sciences study design

All studies must disclose on these points even when the disclosure is negative.

Sample size	In regards to dissected tissues/ brain or head measurements, sample sizes were chosen to be equal to or greater than 5, with numbers being largely dependent on the health of the organism at the developmental stage being tested. In regards to qRT-PCR analyses, hundreds of first instar larvae were pooled together from 4 biological replicates of each genotype, in order to produce significant amounts of RNA for analysis.
Data exclusions	Data was not excluded from this paper.
Replication	Experiments were replicated in at least 2 biological replicates and were reproducible.
Randomization	Other than biological sex, allocation was random.
Blinding	Blinding was not performed. Processing and analysis were independent of sample genotypes.

Reporting for specific materials, systems and methods

We require information from authors about some types of materials, experimental systems and methods used in many studies. Here, indicate whether each material, system or method listed is relevant to your study. If you are not sure if a list item applies to your research, read the appropriate section before selecting a response.

Materials & experimental systems

n/a	Involved in the study	
[ ]	[x]	Antibodies
[x]	[ ]	Eukaryotic cell lines
[x]	[ ]	Palaeontology and archaeology
[ ]	[x]	Animals and other organisms
[x]	[ ]	Clinical data
[x]	[ ]	Dual use research of concern
[x]	[ ]	Plants

Methods

n/a	Involved in the study	
[x]	[ ]	ChIP-seq
[x]	[ ]	Flow cytometry
[x]	[ ]	MRI-based neuroimaging

Antibodies

Antibodies used	anti-deadpan (Abcam ab195173; 1:100)<br><br>anti-GFP (Invitrogen A10262; 1:200)

anti-DCP1 (Cell Signaling 9578; 1:200)

anti-phospho-histone H3 ser10 (Cell Signaling 9701; 1:500)

anti-prospero (Developmental Studies Hybridoma Bank MR1A; 1:1000)

anti-mCherry (Cell signaling 43590; 1:200)

Goat anti-Chicken IgY (H+L) Secondary Antibody, Alexa Fluor™ 488 (Thermofisher A-11039)

Goat anti-Rabbit IgG (H+L) Cross-Adsorbed Secondary Antibody, Alexa Fluor™ 568 (Thermofisher A-11011)

Donkey anti-Rabbit IgG (H+L) Highly Cross-Adsorbed Secondary Antibody, Alexa Fluor™ 647 (Thermofisher A-31573)

Donkey anti-Rabbit IgG (H+L) Highly Cross-Adsorbed Secondary Antibody, Alexa Fluor™ 488 (Thermofisher A-21206)

Goat anti-Mouse IgG (H+L) Cross-Adsorbed Secondary Antibody, Alexa Fluor™ 488 (Thermofisher A-11001)

Goat anti-Mouse IgG (H+L) Cross-Adsorbed Secondary Antibody, Alexa Fluor™ 568 (Thermofisher A-11004)

Goat anti-Mouse IgG (H+L) Cross-Adsorbed Secondary Antibody, Alexa Fluor™ 647 (Thermofisher A-21235)

Validation

All antibodies used within this study are commercially available and verified by the manufacturer to be suitable for immunofluorescence assays. Information regarding the verification of the antibodies and further citations validating the antibody can be found on the company's website:

anti-deadpan (Abcam ab195173) https://www.abcam.com/products/primary-antibodies/deadpan-antibody-11d1bc7-ab195173.html. This antibody was verified for use in immunostaining of Drosophila larval brains and the "Abpromise guarantee" covers the use of the antibody in IHC and IF applications. References can be found at the website listed above.

anti-GFP (Invitrogen A10262) https://www.thermofisher.com/antibody/product/GFP-Antibody-Polyclonal/A10262. This antibody was validated for use in immunofluorescence analyses in human cells and is published for use in Drosophila cells and tissues, as well. This antibody also underwent advanced verification to ensure that the antibody binds to the antigen stated. References can be found at the website listed above.

anti-DCP1 (Cell Signaling 9578) https://www.cellsignal.com/products/primary-antibodies/cleaved-drosophila-dcp-1-asp215-antibody/9578. This antibody was verified for use in Immunofluorescence analyses in Drosophila cells and is quaranteed by the CST Product Performance Guarantee. References can be found at the website listed above.

anti-phospho-histone H3 ser10 (Cell Signaling 9701) https://www.cellsignal.com/products/primary-antibodies/phospho-histone-h3-ser10-antibody/9701. This antibody was validated for use in immunofluorescence analyses in human cells and is published for use in Drosophila cells and tissues, as well. The antibody is quaranteed by the CST Product Performance Guarantee. References can be found at the website listed above.

anti-prospero (Developmental Studies Hybridoma Bank MR1A) https://dshb.biology.uiowa.edu/Prospero-MR1A. This antibody was validated for use in immunostaining of Drosophila larval brains by the Homem lab (images and references on website listed above).

anti-mCherry (Cell signaling 43590) https://www.cellsignal.com/products/primary-antibodies/mcherry-e5d8f-rabbit-mab/43590. This antibody was validated for use in immunostaining analyses in mouse tissues and has also been used for similar experiments in Drosophila tissues. References can be found at the website listed above. The antibody is quaranteed by the CST Product Performance Guarantee.

Goat anti-Chicken IgY (H+L) Secondary Antibody, Alexa Fluor™ 488 (Thermofisher A-11039) https://www.thermofisher.com/antibody/product/Goat-anti-Chicken-IgY-H-L-Secondary-Antibody-Polyclonal/A-11039. To minimize cross-reactivity, these goat anti-chicken IgY whole antibodies have been affinity-purified. Cross-adsorption or pre-adsorption is a purification step to increase specificity of the antibody resulting in higher sensitivity and less background staining. The secondary antibody solution is passed through a column matrix containing immobilized serum proteins from potentially cross-reactive species. Only the nonspecific-binding secondary antibodies are captured in the column, and the highly specific secondaries flow through. The benefits of this extra step are apparent in multiplexing/multicolor-staining experiments (e.g., flow cytometry) where there is potential cross-reactivity with other primary antibodies or in tissue/cell fluorescent staining experiments where there may be the presence of endogenous immunoglobulins. This antibody has been validated for immunostaining in multiple species and references are available on the website listed above.

Goat anti-Rabbit IgG (H+L) Cross-Adsorbed Secondary Antibody, Alexa Fluor™ 568 (Thermofisher A-11011) https://www.thermofisher.com/antibody/product/Goat-anti-Rabbit-IgG-H-L-Cross-Adsorbed-Secondary-Antibody-Polyclonal/A-11011. To minimize cross-reactivity, these goat anti-rabbit IgG whole antibodies have been cross-adsorbed against human IgG, human serum, mouse IgG, mouse serum, and bovine serum. Cross-adsorption or pre-adsorption is a purification step to increase specificity of the antibody resulting in higher sensitivity and less background staining. The secondary antibody solution is passed through a column matrix containing immobilized serum proteins from potentially cross-reactive species. Only the nonspecific-binding secondary antibodies are captured in the column, and the highly specific secondaries flow through. The benefits of this extra step are apparent in multiplexing/multicolor-staining experiments (e.g., flow cytometry) where there is potential cross-reactivity with other primary antibodies or in tissue/cell fluorescent staining experiments where there are may be the presence of endogenous immunoglobulins. Alexa Fluor dyes are among the most trusted fluorescent dyes available today. Invitrogen™ Alexa Fluor 568 dye is a bright, orange/

red-fluorescent dye with excitation ideally suited to the 568 nm laser line. For stable signal generation in imaging and flow cytometry, Alexa Fluor 568 dye is pH-insensitive over a wide molar range. Probes with high fluorescence quantum yield and high photostability allow detection of low-abundance biological structures with great sensitivity. Alexa Fluor 568 dye molecules can be attached to proteins at high molar ratios without significant self-quenching, enabling brighter conjugates and more sensitive detection. The degree of labeling for each conjugate is typically 2-8 fluorophore molecules per IgG molecule; the exact degree of labeling is indicated on the certificate of analysis for each product lot. This antibody has been validated for immunostaining in multiple species and references are available on the website listed above.

Donkey anti-Rabbit IgG (H+L) Highly Cross-Adsorbed Secondary Antibody, Alexa Fluor™ 647 (Thermofisher A-31573) https://www.thermofisher.com/antibody/product/Donkey-anti-Rabbit-IgG-H-L-Highly-Cross-Adsorbed-Secondary-Antibody-Polyclonal/A-31573. To minimize cross-reactivity, these donkey anti-rabbit IgG whole antibodies have been affinity-purified and show a published cross-reactivity to rat IgG. Cross-adsorption or pre-adsorption is a purification step to increase specificity of the antibody resulting in higher sensitivity and less background staining. The secondary antibody solution is passed through a column matrix containing immobilized serum proteins from potentially cross-reactive species. Only the nonspecific-binding secondary antibodies are captured in the column, and the highly specific secondaries flow through. The benefits of this extra step are apparent in multiplexing/multicolor-staining experiments (e.g., flow cytometry) where there is potential cross-reactivity with other primary antibodies or in tissue/cell fluorescent staining experiments where there may be the presence of endogenous immunoglobulins. This antibody has been validated for immunostaining in multiple species and references are available on the website listed above.

Donkey anti-Rabbit IgG (H+L) Highly Cross-Adsorbed Secondary Antibody, Alexa Fluor™ 488 (Thermofisher A-21206) https://www.thermofisher.com/antibody/product/Donkey-anti-Rabbit-IgG-H-L-Highly-Cross-Adsorbed-Secondary-Antibody-Polyclonal/A-21206. To minimize cross-reactivity, these donkey anti-rabbit IgG whole antibodies have been affinity-purified and show minimum cross-reactivity to bovine, chicken, goat, guinea pig, hamster, horse, human, mouse, rat, and sheep serum proteins. Cross-adsorption or pre-adsorption is a purification step to increase specificity of the antibody resulting in higher sensitivity and less background staining. The secondary antibody solution is passed through a column matrix containing immobilized serum proteins from potentially cross-reactive species. Only the nonspecific-binding secondary antibodies are captured in the column, and the highly specific secondaries flow through. The benefits of this extra step are apparent in multiplexing/multicolor-staining experiments (e.g., flow cytometry) where there is potential cross-reactivity with other primary antibodies or in tissue/cell fluorescent staining experiments where there may be the presence of endogenous immunoglobulins. This antibody has been validated for immunostaining in multiple species and references are available on the website listed above.

Goat anti-Mouse IgG (H+L) Cross-Adsorbed Secondary Antibody, Alexa Fluor™ 488 (Thermofisher A-11001) https://www.thermofisher.com/antibody/product/Goat-anti-Mouse-IgG-H-L-Cross-Adsorbed-Secondary-Antibody-Polyclonal/A-11001. To minimize cross-reactivity, these goat anti-mouse IgG whole antibodies have been cross-adsorbed against human IgG and human serum. Cross-adsorption or pre-adsorption is a purification step to increase specificity of the antibody resulting in higher sensitivity and less background staining. The secondary antibody solution is passed through a column matrix containing immobilized serum proteins from potentially cross-reactive species. Only the nonspecific-binding secondary antibodies are captured in the column, and the highly specific secondaries flow through. The benefits of this extra step are apparent in multiplexing/multicolor-staining experiments (e.g., flow cytometry) where there is potential cross-reactivity with other primary antibodies or in tissue/cell fluorescent staining experiments where there are may be the presence of endogenous immunoglobulins. This antibody has been validated for immunostaining in multiple species and references are available on the website listed above.

Goat anti-Mouse IgG (H+L) Cross-Adsorbed Secondary Antibody, Alexa Fluor™ 568 (Thermofisher A-11004) https://www.thermofisher.com/antibody/product/Goat-anti-Mouse-IgG-H-L-Cross-Adsorbed-Secondary-Antibody-Polyclonal/A-11004. To minimize cross-reactivity, these goat anti-mouse IgG (H+L) whole secondary antibodies have been affinity purified and cross-adsorbed against human IgG and human serum prior to conjugation. Cross-adsorption or pre-adsorption is a purification step to increase specificity of the antibody resulting in higher sensitivity and less background staining. The secondary antibody solution is passed through a column matrix containing immobilized serum proteins from potentially cross-reactive species. Only the nonspecific-binding secondary antibodies are captured in the column, and the highly specific secondaries flow through. The benefits of this extra step are apparent in multiplexing/multicolor-staining experiments (e.g., flow cytometry) where there is potential cross-reactivity with other primary antibodies or in tissue/cell fluorescent staining experiments where there are may be the presence of endogenous immunoglobulins. This antibody has been validated for immunostaining in multiple species and references are available on the website listed above.

Goat anti-Mouse IgG (H+L) Cross-Adsorbed Secondary Antibody, Alexa Fluor™ 647 (Thermofisher A-21235) https://www.thermofisher.com/antibody/product/Goat-anti-Mouse-IgG-H-L-Cross-Adsorbed-Secondary-Antibody-Polyclonal/A-21235. To minimize cross-reactivity, these goat anti-mouse IgG (H+L) whole secondary antibodies have been affinity purified and cross-adsorbed against human IgG and human serum prior to conjugation. Cross-adsorption or pre-adsorption is a purification step to increase specificity of the antibody resulting in higher sensitivity and less background staining. The secondary antibody solution is passed through a column matrix containing immobilized serum proteins from potentially cross-reactive species. Only the nonspecific-binding secondary antibodies are captured in the column, and the highly specific secondaries flow through. The benefits of this extra step are apparent in multiplexing/multicolor-staining experiments (e.g., flow cytometry) where there is potential cross-reactivity with other primary antibodies or in tissue/cell fluorescent staining experiments where there are may be the presence of endogenous immunoglobulins. This antibody has been validated for immunostaining in multiple species and references are available on the website listed above.

Animals and other research organisms

Policy information about studies involving animals; ARRIVE guidelines recommended for reporting animal research, and Sex and Gender in Research

Laboratory animals	Drosophila melanogaster (fruitflies) were used in these studies. The ages of flies are specified in the Methods section of the manuscript, and ages varied, depending on the experiment and the question being addressed.<br><br>The following wild type, mutant, and deficiency stocks were used in these studies:<br><br>w[1118] (Bloomington Stock Center 6326), w[1118]; dCap-D3c07081/c07081 (Harvard Exelixis collection generated in the w[1118] line38, w[1118]; Df(2L)Exel7023/CyO (Bloomington Stock Center 7797), w[1118]; Df(3L)BSC558/TM6C, Sb[1] (Bloomington Stock Center 25120), w[1118]; AGO2[454]/TM3, Sb[1] Ser[1] (Bloomington Stock Center 36512)<br><br>The following RNAi stocks were used in these studies:<br><br>UAS-GFP dsRNA (P{UAS-GFP.dsRNA.R}142; Bloomington Stock Center 9330), UAS-cap-d3 dsRNA (w1118; P{GD913}; VDRC stocks 29657 and 9402), UAS-smc2-dsRNA (P{KK100466}VIE-260B; VDRC stock 103406 and P{TRiP.GL00440}attP40; Bloomington Stock Center 35602), UAS-cap-d2 dsRNA (P{KK101115}VIE-260B; VDRC stock 108289 and w1118; P{GD9677}v33424 ; VDRC stock 33424), UAS-cap-h dsRNA (y1 sc* v1 sev21; P{TRiP.HMS00049}attP2; Bloomington Stock Center 34068).<br><br>The following GAL4 and UAS stocks were used in these studies:<br><br>armadillo GAL4 (w[*]; P{w[+mW.hs]=GAL4-arm.S}4a P{w[+mW.hs]=GAL4-arm.S}4b; Bloomington Stock Center 1561 that has lost the balancer), eyGAL4, GMRGAL4 (generous gift from Dr. Bob Johnston at Johns Hopkins University), UAS-GFP.nls (P{UAS-GFP.nls}14; Bloomington Stock 4775), UAS-GFP-CAP-D339, tubPGAL4 (Bloomington Stock 5138), repoGAL4 (generous gift from Dr. Heather Broihier at Case Western Reserve University), elavGAL4 (Bloomington Stock Center 8765), c253GAL4 (w[1118]; P{w[+mW.hs]=GawB}C253; Bloomington Stock Center 6980), dpnGAL4 (Bloomington Stock Center 47456), optixGAL4 (w[1118]; P{y[+t7.7] w[+mC]=GMR30D11-GAL4}attP2; Bloomington Stock Center 48098), UAS-gypsyCLEVR and UAS-gypsy-CLEVRΔPBS
Wild animals	No wild animals were used in this study.
Reporting on sex	Both biological sexes were considered in these studies. For all experiments, the biological sex of the adults, pupae, or larvae is listed. For some experiments, it was not possible to perform experiments using both sexes, since the indicated genotypes resulted in lethality.
Field-collected samples	No field collected samples were used in this study.
Ethics oversight	All studies using transgenic Drosophila melanogaster stocks were approved under Cleveland Clinic Institutional Biosafety Committee protocol #1106.

Note that full information on the approval of the study protocol must also be provided in the manuscript.

Plants

Seed stocks	N/A
Novel plant genotypes	N/A
Authentication	N/A
